# Surrogate strains of human pathogens for field release

**DOI:** 10.1080/21655979.2017.1349044

**Published:** 2017-07-26

**Authors:** Sangjin Park, Chang-Hwan Kim, Seong Tae Jeong, Sang Yup Lee

**Affiliations:** a Metabolic and Biomolecular Engineering National Research Laboratory, Department of Chemical and Biomolecular Engineering (BK21 Plus Program), Center for Systems and Synthetic Biotechnology, Institute for the BioCentury, Korea Advanced Institute of Science and Technology (KAIST), Daejeon, Republic of Korea; b The 5th R&D Institute, Agency for Defense Development (ADD), Daejeon, Republic of Korea

**Keywords:** field release, genetic engineering, pathogen, surrogate strain, synthetic biology

## Abstract

Surrogate microorganisms, in short surrogates, are an essential part of pathogen research. Compared to surrogates used in controlled laboratory environments, surrogates for field release are restricted by concerns about human and environmental safety. For field research of food-borne pathogens, strains of an attenuated pathogen or strains of genetically close non-pathogenic species have been used as surrogates. Genetic modification is usually performed to attenuate virulence, through for examples deletion of genes of virulence and transcriptional regulators and removal of virulence plasmids, and to facilitate detection and monitoring through observing antibiotic resistance, fluorescence, and bioluminescence. For field research of a biological warfare agent *Bacillus anthracis*, strains of genetically close non-pathogenic species or strains of genetically distant non-pathogenic species have been used, mostly without any genetic modification. Recently, we constructed strains of *Bacillus thuringiensis* as surrogates for *B. anthracis*, demonstrating that strain engineering could significantly enhance the utility of surrogates, and that the application of a simple genetic circuit could significantly impact surrogate safety. Thus far, enormous potential of biotechnology has not been exploited enough due to safety concerns regarding the field release of genetically engineered microorganisms. However, synthetic biology is rapidly developing, providing new concepts for biocontainment as well as ingenious genetic circuits and devices, which should be applied in future research of field-use surrogates.

## Introduction

Surrogate bacteria are bacteria used in research in the place of a target pathogen, and their use has been essential part of pathogen studies since the early days of microbiology. For best test results, use of the pathogen is desirable, but in most cases, this is restricted due to safety concerns. For biological warfare agents, research is restricted not only by safety requirements, but also by laws and strict regulations. The use of surrogates somewhat relieves researchers from human and environmental safety concerns and facilitates their research works as the production of surrogates is easier and not under strict regulation. Most surrogate research is performed in laboratories, but research in agriculture, food industry, and biodefense requires environmental studies involving field release, where strains cannot be controlled and many experimental variables exist. Due to concerns about human and environmental safety, many laboratory surrogates are not permitted for field use.

For field studies, surrogate strains or parental strains for development of surrogate strains generally fall into 3 categories: naturally or artificially attenuated strains of a target pathogen, strains of genetically close non-pathogenic species, and strains of genetically distant non-pathogenic species. Attenuated strains of a target pathogen will most closely mimic the properties of the pathogen. However, genetically close strains are likely to have similar biological characteristics to a target pathogen. As these strains are not derived from pathogens, they are safer and more likely to get public approval. For this reason, many genetic relatives of pathogens have been used as surrogates. It should be remembered, however, that careful characterization of each candidate strain is essential as genetic closeness does not necessarily guarantee similar biological characteristics.^1,2^ Thus, it is desirable to thoroughly characterize candidate strains, as exemplified in a study of *Salmonella* surrogate construction.^3^ Genetically distant non-pathogenic surrogates are chosen because of their safety profiles and the ease of use and production. As they are genetically distant from target pathogens, their biological characteristics are likely to be different. However, depending on the purpose of the study, different characteristics between the target pathogen and a surrogate need to be considered. For example, as the spore sizes of *B. atrophaeus* and *B. subtilis* are substantially different from *B. anthracis*, they are not suitable as surrogates for aerodynamic studies.^4^ Their sensitivity to chemical disinfecting agents is, however, similar to *B. anthracis*, permitting their use as surrogates in decontamination studies.^5^

In this paper, we will briefly examine various surrogate strains of food-borne pathogens and biological warfare agent *B. anthracis*. The scope of this paper covers surrogate strains actually used for field release, and surrogate strains constructed for field release that might have not been released yet. Here, ‘field release’ means ‘release outside the laboratory’, which can include release in a building or an airplane as well as release to the outdoor environment. Focuses will be given on the strains and their construction. Readers are encouraged to consult the cited studies for further details.

### Surrogate strains for food-borne pathogens

*Escherichia coli* O157:H7 strain ATCC 700728, lacking 2 major toxin genes (*stx_1_* and *stx_2_*), is categorized as a biosafety level 1 bacterium. This strain was used to study the survival of *E. coli* O157:H7 in field-inoculated lettuce.^6^ A spontaneous rifampicin-resistant mutant was isolated after exposure to the antibiotic, and used to facilitate bacterial enumeration.

*E. coli* O157:H7 strain 3704, a naturally nontoxigenic strain, was marked with bioluminescence by transposon mutagenesis with the *luxCDABE* cassette from *Photorhabdus luminescens* to evaluate long-term survival in the environment.^7^ By introducing the cassette into the chromosome, the *lux* phenotype was maintained stably without any antibiotics. As bioluminescence is linked to cellular metabolic activities,^8^ bioluminescence assays can measure the active population and metabolic status of cells in the study.

A green fluorescent protein (GFP) expression plasmid was introduced into 4 non-toxigenic strains of *E. coli* O157:H7, which naturally lacked *stx_1_* and *stx_2_*, to study internalization of the pathogen into field-grown vegetables.^9^ The GFP plasmid was maintained with ampicillin during cultivation. Bacterial enumeration was performed on tryptic soy agar (TSA) plates supplemented with ampicillin, and GFP fluorescence was used to identify correct colonies. Although antibiotic resistance is often used for identifying colonies in surrogate studies, many microorganisms in soil can show natural resistance to the antibiotics used in the experiment. The use of GFP fluorescence could ensure correct identification of surrogate colonies.

To address concerns that naturally non-toxigenic strains and toxigenic strains might differ in biological characteristics, 2 toxigenic strains isolated from actual outbreaks were used as parental strains for surrogate construction.^10^ Shiga toxin genes as well as the *eae* virulence-related gene were deleted. In addition, a GFPuv plasmid was transformed into the strains for easy detection. Bacterial enumeration was performed on TSA plates supplemented with ampicillin, and GFPuv fluorescence was used to identify correct colonies.

A vaccine candidate strain of the *Salmonella enterica* serovar Typhimurium (*S*. Typhimurium) chi 3985 was used to study the persistence of *Salmonella* in soil and on vegetables after use of contaminated compost and irrigation water.^11^ This strain was made from the virulent *Salmonella* chi 3761 strain by deleting 2 genes, *cya* (adenylate cyclase) and *crp* (cyclic AMP receptor protein), by transposon mutagenesis.^12^ A cyclic AMP receptor protein, when bound by cAMP (cyclic AMP) produced by an adenylate cyclase, acts as a transcriptional regulator of many genes.^13^ The deletion of these genes caused the strain to be avirulent, though the mechanism of this attenuation has not been fully elucidated in *Salmonella*.

As an attenuated strain such as chi 3985 retains intact virulence genes on its chromosome, the avirulent *Salmonella* Typhimurium MHM112 strain was constructed by removing virulence-related genes and a plasmid from the virulent strain 14028.^3^ This strain was constructed to model *Salmonella* behavior in the environment. Five *Salmonella* pathogenicity islands and the virulence plasmid pSLT were removed to make it avirulent. As the λ Red system with flippase (Flp) recombinase was used, no antibiotic markers or any foreign genes (except Flp recognition targets) were left on the chromosome. A *phoN* mutation was introduced to make the strain easily distinguishable from the wild-type strain using a chromogenic substrate. Extensive characterization of biological characteristics was performed, and the results supported its suitability as a surrogate for environmental studies.

*Listeria innocua* and *Clostridium sporogenes* are categorized as biological safety level 1 bacteria, and are popularly used as surrogates for *Listeria monocytogenes* and *Clostridium botulinum*, respectively. *L. innocua* stain CIP 80–12 and *C. sporogenes* strain CIP 79–3 were used as surrogates to monitor their behavior during parsley production in fields.^14^ For enumeration of bacteria, a selective agar (Oxford agar) and a differential agar (Differential Reduced Clostridial Medium) were used for *L. innocua* and *C. sporogenes*, respectively. Before field tests, *L. innocua* CIP 80–12 was demonstrated to survive better than 2 strains of *L. monocytogenes* in soil microcosms in the laboratory, proving its suitability as a surrogate.

### Surrogate strains for the biological warfare agent *B. anthracis*

Many differences exist between surrogates for food-borne pathogens and those for *B. anthracis* (Table 1). For food-borne pathogens, many avirulent or attenuated pathogens are used as surrogates or as parental strains for surrogate construction. For *B. anthracis*, this rarely happens, due to the risk of genetic exchange (e.g., horizontal gene transfer) in the environment, lack of public acceptance, and the possibility of false positive anthrax detection due to remaining surrogate spores.^15^ Most studies using attenuated strains of *B. anthracis* as surrogates are performed inside laboratories with safety equipment.

**Table 1.** Comparison between field-use surrogates of food-borne pathogens and *B. anthracis*.

	food-borne pathogen surrogates	*B. anthracis* surrogates
Purpose	• To evaluate agricultural practices	• To simulate biologic attack
	• To analyze sources of contamination in the field	• To evaluate response performance (detection and decontamination)
	• To study persistence and behavior of pathogens in the field	• To analyze dissemination and dispersion
Related areas	Agriculture, food industry	Biodefense
Form of surrogate release	Vegetative cell	Spore
Method of release	Inoculation in vegetables, water, compost, and soil	Mostly aerosol release
Inhalation risk during test	Low	Very high
Use of antibiotic resistance to facilitate bacterial enumeration	Often	No
Persistence	Months	Years, sometimes decades
Surrogate strains or parental strains for surrogate	• Naturally or artificially attenuated strains of a target pathogen	• Strains of genetically close non-pathogenic species
construction	• Strains of genetically close, non-pathogenic species	• Strains of genetically distant non-pathogenic species
Detection method	• Growth on selective media (often with antibiotics)	• Growth on general rich media
	• Fluorescence with GFP	• Growth on selective media
	• Bioluminescence with *lux* genes	• PCR (often real-time PCR)
Engineering^a^	• No engineering in many cases	• No engineering in most cases
	• Deletion of virulence-related genes	• *cry* plasmid curing
	• Removal of virulence plasmid	• Genetic barcode insertion
	• Deletion of transcriptional regulation-related genes	
	• Screening for spontaneous antibiotic-resistant mutants	
	• Transformation of GFP plasmid (with an antibiotic marker) for easy detection	
	• Insertion of *lux* genes for monitoring	

a The result of our study (see ref. 26) is not included.

*Bacillus atrophaeus* (traditionally known as *Bacillus globigii*) has been the most popular surrogate of *B. anthracis*.^15,16^ It is a spore-forming bacterium with high spore yield, and is soil dwelling and non-pathogenic (biosafety level 1). All these properties make *B. atrophaeus* a popular surrogate for studies of anthrax spores, although it is genetically distant from *B. anthracis* (see ref.16) and has a smaller spore size.^4^ It was used to study migration of spores during the decontamination of a 2-story building (see ref.17) and to study dissemination of spores and mitigation measures in simulated anthrax letter attacks within an office building.^18^

*Bacillus amyloliquefaciens* was released to simulate a terrorist attack and evaluate detection performance in an urban area.^19^ *B. amyloliquefaciens* is a close relative of *B. subtilis*, and its history of use as biopesticide ensures safety. It was assumed that detection of *B. amyloliquefaciens* was easier than other *Bacillus*-based pesticides, as it is less abundant in the environment. A commercial biopesticide containing spores of the strain was released as a dry powder in the city.

*Bacillus thuringiensis* was proposed as a more suitable surrogate for *B. anthracis*, since it is a genetically close relative with similar biological characteristics, and has a well-known safety profile with long history of its use as biopesticide.^2,15^ Spores of *B. thuringiensis* var. kurstaki ABTS-351 were used as surrogates for *B. anthracis* in 2 studies. This strain has been used as a bioinsecticide against gypsy moths, and is safe for humans and the environment. A commercial bioinsecticide containing the spores of the strain was sprayed aerially in urban areas to study transport of the surrogate via formites.^20^ The same bioinsecticide was aerially sprayed in urban areas to study transport of the surrogate into buildings after outdoor release.^21^

### Development of *B. thuringiensis* as a surrogate for *B. anthracis*

The studies mentioned above used *B. thuringiensis* without any genetic modification. So far, 3 studies have developed strains of *B. thuringiensis* to improve its properties as a surrogate for *B. anthracis*. In the first study,^22^ the parental strain used was *B. thuringiensis* HD-1. With a long history of its use as a bioinsecticide worldwide, the HD-1 strain has an excellent safety profile. Engineering was performed on this strain to solve problems associated with repetitive use of the surrogate in fields. Due to the long-term persistence of spores, it is difficult to conduct tests with the same strains on the same test site several times; this is because the previously used spores persist and interfere with those used in the next tests. To overcome this problem, a genetic barcode, consisting of common and specific 20-bp tags, was inserted into carefully selected sites on the chromosome. An I-SceI meganuclease-based system was used during strain construction, ensuring that no antibiotic marker remained. The inserted barcode was very stable, with no loss over 300 generations, and no significant difference in growth was observed between the barcoded and wild-type strains. The barcodes were detected by real-time polymerase chain reaction (PCR). The common tag was used to differentiate the constructed strains from the wild-type strain or other strains of *B. thuringiensis*, while the specific tag was used to differentiate constructed strains from each other. As tagged strains are identical to each other except for the specific tags, they can be used on the same test site under different conditions. The utility of this work was confirmed by indoor tunnel test and open-air field release test.^23^

In the second study,^24^ 2 isolates of *B. thuringiensis* HD-1 were cured of *cry*-bearing plasmids, which were present in the HD-1 strain of the first study,^22^ by culturing at high temperature (42°C). The plasmid-cured strains did not produce Cry insecticidal proteins, which are necessary for their bioinsecticidal function, but not for their use as surrogates. As no genetic tool was used, these strains would not be regarded as a genetically modified organism; therefore, it might be easier to get environmental release studies approved from regulatory agencies. These strains were used together with spores of *B. thuringiensis* Al Hakam as surrogates for *B. anthracis* to study a method of decontamination in C-130 aircraft.^25^

In the third study we recently reported,^26^ *B. thuringiensis* surrogate strains were constructed using the most extensive engineering to date. *B. thuringiensis* BMB171 was used as a parental strain because it has high transformation efficiency and no Cry-producing plasmids.^27^ It was aimed to make *B. thuringiensis* a more suitable surrogate, with increased ease of detection and enhanced safety for humans and the environment. As many spore-forming bacteria in soil have colony morphologies and colors similar to *B. thuringiensis*, the *crtM-crtN* genes from *Staphylococcus aureus* were introduced to confer yellow colony color. Since the *crtM-crtN* carrying plasmid can be easily cured under no selective pressure (e.g., antibiotics) in environment, these genes were inserted into the chromosome by transposon delivery vector. The engineered strain produced the yellow pigment 4,4′-diaponeurosporene, which made colonies easily distinguishable from colonies of other soil bacteria. For increased environmental safety, a simple genetic circuit, which deleted the sporulation master regulator gene *spo0A* during the sporulation process, was constructed. Introduction of this circuit into the strain made cells that produce spores without further sporulation ability. This circuit comprised the *spo0A* gene surrounded by 2 *loxP* sites, and the Cre recombinase gene expressed by a sporulation-dependent promoter, which was inserted at another locus on the chromosome. During sporulation, Cre recombinase is expressed and the *spo0A* gene is deleted. As *spo0A* is required for the initiation of sporulation, these spores cannot enter sporulation process again. The survival of *B. thuringiensis* in soil is severely diminished by *spo0A* deletion,^28^^,^^29^ and consequently this circuit is expected to decrease the persistence of surrogate spores. Lastly, 2 major genes encoding α/β-type small acid-soluble spore proteins (SASPs; SspA and SspB) were deleted. This deletion markedly increased the spore's sensitivities toward UV-C, temperature, and artificial sunlight, which is expected to diminish the environmental persistence of the spores after release. For persistence studies, these features might not be appropriate, but this enhanced environmental friendliness would certainly be an advantage for studies where environmental persistence is not required or desirable. Moreover, the deletion of these genes can substantially contribute to the enhancement of human safety, since spores of the mutant strain, when delivered intratracheally, were quickly cleared from the lungs of mice, whereas spores of the wild-type strain persisted long consistently with a previous report.^30^ Flp recombinase and I-SceI meganuclease were used for the above strain construction, leaving no antibiotic markers on the chromosome. This work was the first report of an attempt to control the persistence of surrogate spores for field release through genetic engineering. However, whether all these features work as expected on field release remains to be elucidated.

### Future prospects

**Figure 1.**
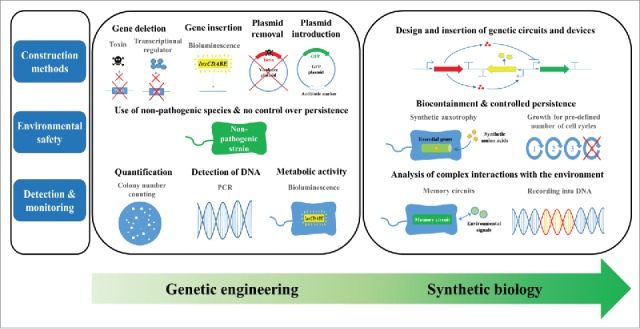
Current status and future prospects of research for field-use surrogates. Current surrogate construction primarily depends on simple deletion (removal) and insertion, while design and insertion of genetic circuits and devices in synthetic biology would potentially expand utility of surrogates in the future. Concerns regarding environmental release of genetically engineered microorganisms can be overcome by biocontainment strategies of synthetic biology, whereas current surrogate research has solely relied on natural decay of non-pathogenic microorganisms after release. In the coming years, synthetic biology would enable analysis of more complex interactions between the surrogates and environment beyond those currently studied through rather simple analyses, such as counting the number of colonies, PCR-based DNA detection, and measuring bioluminescence.

Advanced strain engineering techniques developed over the years have been little exploited for the development of field-use surrogates. Even application of a simple genetic circuit, as in our study, has been rare. Potential environmental impacts of genetically engineered microorganisms remain the primary obstacle associated with their environmental release studies.^31^ So far, synthetic biology has had little impact on surrogate research, despite its tremendous potential to reshape surrogate construction (Fig. 1). However, synthetic biology is beginning to provide new approaches for biocontainment.

The use of auxotrophs has been long proposed as a good biocontainment strategy; however, the requirement for an additional nutrient can be circumvented by nutrients in environment.^32^ To solve this problem, synthetic auxotrophy was proposed. Strains of *E. coli*, growth of which was dependent on synthetic amino acids, were constructed based on a genomically recoded organism (GRO) lacking all TAG stop codons plus release factor 1.^33^ Using an orthogonal translation system, the TAG codon was changed into a sense codon for synthetic amino acids, which were incorporated into essential genes. As the synthetic amino acids cannot be found outside the laboratory, complementation from other sources in nature is almost, if not all, impossible. When multiple essential genes were targeted for TAG codon incorporation, an escape frequency below 6.3 × 10^−12^ was achieved. In addition to synthetic auxotrophy, many novel biocontainment approaches are being developed by synthetic biologists.^32,34^ Although biocontainment is not appropriate for persistence studies, depending on the purpose and type of the study, biocontainment could be applicable. When properly done, it will allow outdoor use of engineered surrogates much more safely and comfortably.

Biocontainment is not the only area where synthetic biology can contribute to developing surrogates. Many ingenious circuits and devices can add useful, previously inconceivable features to surrogate strains. For example, many ‘kill switches’ can be combined with temperature sensors, making strains that survive only above (or below) a certain temperature. Growth of such surrogates will be restricted to a specific season, so new rounds of testing can begin each year. Synthetic gene networks that allow counting of the number of events can be used to make a surrogate that grows only for a pre-defined number of cell cycles.^35^ For example, if this circuit is combined with our sporulation-dependent *spo0A* knockout circuit, it would be possible to make a *Bacillus* strain that can form spores for a predefined number of times, instead of once as demonstrated in our study. This kind of controlled persistence will be very useful for certain types of surrogate research.

Currently, most post-release analysis has focused on the quantification of cells (through counting colony forming units) or DNA (through PCR). Analysis of how surrogates interact with the environment is rarely accomplished, as this kind of analysis is very difficult to perform. Kotula et al. (see ref.36) reported a genetic circuit that enabled *E. coli* to remember an environmental signal. When cells of this *E. coli* strain were given to mice orally, the recovered bacteria remembered *in vivo* experiences with a given environmental signal. If this genetic circuit is applied to surrogate strains, it will be possible to make surrogates that can report their interactions with the environment after field release. Synthetic biologists are also developing bacteria that can record their memories directly into genomic DNA.^37,38^ Thus, surrogate strains can be equipped with a genetic recording circuit linked to activation of specific transcriptional regulators in response to environmental perturbations, such as exposure to toxic chemicals, antibiotics, or sudden pH change. Then, surrogate strains can operate the synthetic circuits to remember such perturbations and report back to us. With further advances in this research, surrogates that can remember and report complex environmental experiences might be possible.
